# Facilitating the commercialization and use of organ platforms generated by the microphysiological systems (Tissue Chip) program through public–private partnerships

**DOI:** 10.1016/j.csbj.2016.04.003

**Published:** 2016-05-10

**Authors:** Christine A. Livingston, Kristin M. Fabre, Danilo A. Tagle

**Affiliations:** National Center for Advancing Translational Sciences, National Institutes of Health, Bethesda, MD 20892, United States

**Keywords:** Tissue Chip, Commercialization, Microphysiological systems, Organs-on-chips, Public–private partnerships, RFI: Request for information

## Abstract

Microphysiological systems (organs-on-chips, tissue chips) are devices designed to recapitulate human physiology that could be used to better understand drug responses not easily addressed using other in vivo systems or in vitro animal models. Although still in development, initial results seem promising as tissue chips exhibit in vivo systems-like functional responses. The National Center for Advancing Translation Science (NCATS) identifies this technology as a potential tool that could improve the process of getting safer, more effective treatments to patients, and has led to the Tissue Chip Program, which aims to develop, integrate and validate major organ systems for testing. In addition to organ chip development, NCATS emphasizes disseminating the technology to researchers. Commercialization has become an important issue, reflecting the difficulty of translation from discovery to adoption and wide availability. Therefore, NCATS issued a Request for Information (RFI) targeted to existing partnerships for commercializing tissue chips. The goal was to identify successes, failures and the best practices that could provide useful guidance for future partnerships aiming to make tissue chip technology widely available.

Primary obstacles to the development of new therapeutic drugs are the significant time and resources required to identify and refine new compounds, the lack of better in vitro and in vivo models that are able to accurately predict the safety and efficacy of candidate therapeutics in humans, and submission of the data needed for Food and Drug Administration (FDA) regulatory approval [1], [2], [3]. The needed capital investment, coupled with high failure rates in clinical trials, makes pharmaceutical development high risk [3], [4], [5]. Many compounds ultimately fail in clinical trials due to toxicity or lack of efficacy in humans that is not evident in preclinical data from in vitro or animal testing [6]. To increase the rate at which promising compounds are identified and to reduce the number of compounds that fail in costly and time-consuming clinical trials, the National Center for Advancing Translational Sciences (NCATS) at the National Institutes of Health (NIH) in collaboration with the Defense Advanced Research Projects Agency (DARPA) and the FDA, initiated the NIH Tissue Chip for Drug Screening Program to develop human microphysiological organ systems (MPS) for toxicity and efficacy testing.

The Tissue Chip (TC) Program is to develop human tissue or organ systems (e.g., heart, liver, kidney, nervous system) on bioengineered platforms (i.e., “chips”) [7]. The platforms support the key functional elements (such as 3D tissue architecture, multi-cellularity, biomechanical transduction properties, etc.) of organs under conditions that mimic the physiological and mechanical environment found in vivo, and are designed to facilitate functional readouts (e.g., cardiac contractility, gene expression) [4], [8], [9], [10], [11], [12], [13], [14]. A long term objective is to integrate multiple organ system platforms into a “human on a chip” for a more comprehensive evaluation of drug toxicity and efficacy (Tissue Chip-Integration) [14], [15], [16], [17]. The development and integration of “organs on chips” requires multidisciplinary collaboration of basic scientists, clinicians and bioengineers [10], [11], [13], [14], [18]. These collaborations have been facilitated greatly by productive public–private partnerships that advance the interests of both sectors. To foster additional partnerships and to identify factors contributing to their success, NCATS issued a Request for Information (NOT-TR-14-008, *Public–Private Partnerships for Organ Systems and Platforms Developed by Microphysiological Systems* (*MPS*) *Investigators*). In this report we summarize the responses, which reveal not only factors contributing to success, but also highlight broad interest in the further development, adoption and availability of the tissue chip technology for pharmaceutical development and research purposes.

## 1 Characteristics of successful public–private partnerships

Many partnerships within the TC Program include academic and private sector scientists with mutual interests in the development of the organ systems and in advancing the design of the bioengineered platforms. In some cases, NIH and DARPA-funded academic scientists have contributed biological expertise and provided feed-back related to design requirements, while private sector participants contributed engineering expertise, production efficiency and marketing experience. The collaborative aims of the public and private sectors are further refinement and marketing of the microphysiological organ systems and platforms as relevant research and testing tools. Participants in productive partnerships that achieve these aims consistently cite certain factors that contribute to their success: 1) opportunities for balanced contributions and mutual benefits for both the private and public participants; 2) an organized leadership team with well-defined roles; 3) effective communication between basic scientists, engineers and senior members of the leadership team; 4) productive exchange with potential stakeholders; 5) early identification of a target market; and 6) early consideration of collaborative agreements that address intellectual property issues.

The most productive TC public-private partnerships are based on balanced contributions and subsequent benefits for all. Most importantly, public and private partners first need to identify and align their goals. Additional approaches to balanced contributions are discussed below. The benefits for public and private partners are more rapid scientific development and adoption of the organ system platforms, with the potential for scientific advances and financial profit. The most successful partnerships may lead to widespread use of tissue chips by the pharmaceutical industry, adoption for toxicology testing, and translation for clinical applications (e.g., personalized or precision medicine).

Optimally, partnerships are built on an organized leadership team with each member having a well-defined role. Experienced individuals serving in leadership roles can articulate objectives clearly and focus team efforts. The most efficient partnerships include a small group of select individuals serving as team leaders who can identify primary objectives, integrate them, and direct development. Proximity of leadership and private sector partners to basic and clinical scientists is advantageous in the early stages of development, as this facilitates discussion, testing, access to clinical resources, and can eliminate “middlemen.” Ongoing feedback from private sector partners based on their knowledge of manufacturing and marketing is critical for later stages of development. The integration of this feedback and that from publically funded biomedical scientists is essential to achieving long-term objectives.

Effective communication between publically supported TC scientists and private sector partners is necessary to assure both scientific validity and that organ system platforms will meet market needs. Early identification of the key features required and goals for use of the platforms is essential for efficiency and aggregate solutions, leading to well integrated design. The functional read-outs that are needed should be identified ahead of time, ideally with input from public, private and regulatory sectors. Platform design is driven in part by requirements for validation, but also by the organ systems' and/or platforms' intended use(s). They may be designed for a narrowly targeted market, or allow for modifications to meet the needs of a broader market. For example large pharmaceutical companies may have a wide variety of applications for ex vivo organ systems, dictating that a range of platform options be available: they may need 2- or 3-dimensional systems, single or paired organ system platforms, or multi-organ integrated platforms. Design priorities that consider the scientific validity of the organ systems while balancing market demand should be determined early in development.

In addition to communication between scientists and engineers partnering to develop and/or market the tissue chips, valuable input may come also from stakeholders and early consumers. Therefore, during early translation of the organ systems and platforms to consumers, it is advantageous to anticipate an extended period of technical support and discussion. This facilitates closer collaboration between engineers and biologists, and between developers and consumers, leading to more rapid improvements in design and assurance that the devices meet requirements. Importantly, private sector partners should be prepared to provide a high level of technical support, particularly in the early marketing phase.

As a target market is identified, demonstrating the profit potential may be necessary to attract investors. These markets may include the pharmaceutical/biotechnology, research or clinical sectors. The pharmaceutical or biotechnology sectors, acting as service providers, are likely to use the organ systems and/or platforms for preclinical assays. This may be the most profitable market initially and therefore likely to attract the largest capital investment. Alternatively, the research sector may implement the organ systems and/or platforms as tools. This sector may represent numerous users, but is likely to provide a lower profit margin and may be more attractive to smaller investors. The clinical sector, using the organ system platforms for diagnostics and individualized medicine applications, may be limited initially, but has much potential for growth. Until an organ system platform is validated and progresses towards regulatory qualification, this market may attract limited financial support. Larger investments in products targeting the clinical sector may not occur until development by smaller biotechnology firms has progressed far enough to establish a viable clinical market. Notably, the features of the organ system platforms required by each of these sectors will be different and may dictate shifts in design and development as each market comes to the forefront. Parallel development of platforms for different uses and multiple markets should be feasible.

In parallel with the scientific development and engineering of the organ system platforms, it is imperative that both academic and private sector partners give early consideration to legal issues and to a business plan. These should be addressed early to avoid later complications that would delay scientific progress or the broader distribution of this novel technology. For example, provisions for confidentiality and intellectual property must be agreed upon in the early stages of development, in consultation with institutional and corporate legal representatives. Agreements should address issues such as patenting, licensing arrangements, revenue sharing, and when appropriate, NIH resource sharing requirements. Once a good legal plan is established, it can serve as a general model for future partnership agreements.

## 2 Regulatory considerations

A key incentive for private sector participation and investor interest is regulatory qualification and validation of the organ system platforms. Validation processes will depend on the intended use of the TCs, either as a stand-alone approach (e.g., precision medicine) or as one element in an integrated set of approaches (e.g., dose–response, toxicity and efficacy testing) [4], [14], [15], [16]. The TCs may be used as a tool for mechanistic studies with a reduced number of variables [12]; they may be developed for preliminary or efficient dose–response testing for efficacy and off-target effects, thus reducing the cost of drug development [19]; or TCs developed from human cells can serve as a preclinical approach to identify compounds whose efficacy or toxicity is different in animal vs. human tissue [4], [8], [9], [20]. As an example of the latter, TCs can illustrate significant differences in hepatotoxicity in animals vs. humans [21], [22].

Validation of human organ chips for any use will be a complex process, requiring a long term commitment to the goal of the FDA qualifying the MPS as a valid research tool. Ultimately, validation will require a comparison of the toxicity and efficacy data for compounds tested in preclinical animal studies, using the human tissue chip platforms, and in clinical trials. Demonstration that the results of testing using human microphysiological organ systems parallel those from clinical trials is critical for regulatory approval. Demonstration that the TC platforms can be used to identify toxic or ineffective compounds that have failed in human clinical trials despite promising preclinical data from animals would incentivize the market and investors.

Short term goals for validation can be defined to initiate regulatory consideration of tissue chip technology. As the organ systems and related tools are refined, input and guidance from the FDA will be needed; in parallel, participation by the pharmaceutical industry will also help to determine specifications and to identify biomarkers for which FDA approvals are needed. Several approaches are likely to stimulate input from the FDA. First, promoting more widespread use of the TC platforms, so that data from them begin to appear more regularly in FDA applications will lead to greater familiarity and understanding of the tissue chip technology. The submission of reliable data from TCs will generate more confidence in this new technology. This may not be adequate as “stand alone” evidence: initially, data from MPS may be most effective when submitted as complementary to other models. High quality data from MPS that are consistent with that derived from other approaches will provide initial evidence for the reliability of this new technology, and will allow agencies to evaluate the utility and current limitations of this approach. Importantly, the submission of data derived from MPS to regulatory agencies can support the use of this technology to answer questions that cannot be addressed using animal models. For example, TCs may be particularly useful for determining the mechanism of drug action and relevance in human systems.

This leads to a second approach for soliciting input from regulatory agencies such as the FDA. As part of its Critical Path program, the FDA developed the Biomarker Qualification Program (Drug Development Tools – Biomarker Qualification Program); validation of individual biomarkers on individual chips may be a key step towards qualification and acceptance of tissue chips. Qualification may result in FDA clearance to market the tissue chip technology as a tool for therapeutics development; this could occur long before FDA acceptance of the technology as a clinical diagnostic or treatment tool (e.g., individualized medicine). Importantly, consortia such as the TC Program could be an important element in biomarker qualification applications. Thirdly, immediate use of the tissue chip technology may be pursued via the FDA's Investigational New Drug (FDA Investigational New Drug [IND] Application) or New Drug Application (New Drug Application [NDA]) procedures; approval via these pathways will require investigators' documentation and validation of key biomarkers that focus on well-known pathologies (e.g., arrhythmia, contractility, troponin release). Lastly, the NIH and TC Consortium could consider working with the FDA via its Broad Agency Announcement (BAA)/Program for Extramural Regulatory Science and Innovation (PERSI; FDA Program for Regulatory Science and Innovation). Several of the TC Program objectives coincide with the regulatory research areas targeted for advanced development by the FDA's PERSI: improved toxicological approaches to increase therapeutic safety; innovative approaches to clinical evaluation to improve product development and personalized medicine for improved patient outcomes; and improved approaches to the evaluation of therapeutics' quality.

## 3 Roles of the NIH and Tissue Chip Consortium

In response to the question of what the NIH and NCATS can do to facilitate TC Investigators' progress and the development of strategic public–private partnerships, respondents cited two roles. First, NCATS might coordinate the identification of tools, resources and protocols that are common requirements for TC Investigators. The TC consortium has the potential to leverage members' collective data for consensus-based solutions and device-development, and might play an important role in identifying reliable sources for common requirements and in facilitating access to them.

Among the requirements for the TC Program is a reliable source of cells, in particular human induced pluripotent stem cells (hiPSC), to serve as a renewable tissue source from which the organs on chips can be developed. Compiling input from TC investigators, NCATS might assist in setting standards for the characterization of and reliable access to hiPSC for investigators who wish to adopt the tissue chip technology. Standardized differentiation protocols also may be useful. Collectively reliable access, careful hiPSC characterization and optimized differentiation protocols will ensure reproducibility, thus contributing towards standardization of the tissue chips and making them a more attractive technology for research and testing laboratories. A collaborative approach towards these requirements has proven extremely valuable in developing single organ systems. The additional complexities of multi-organ integration (i.e., multiple tissue types and differentiation protocols) would present a significant hurdle to adoption of the organ platform technology by any single entity, in particular those with limited resources for research and development. A consortium of investigators with broad expertise could address these complex problems more efficiently.

A contract awarded to a private sector company or contract research organization may be one way to ensure availability of a repository of cells appropriate for the tissue chips. Important considerations would be the contract recipient's responsibility for: 1) maintaining a repository of well-characterized, cryopreserved cells, 2) their proliferation, 3) differentiation, and 4) distribution. The repository should include cell lines and hiPSC that have been used widely by the pharmaceutical or research sectors; these cells are characterized already and prior data sets are available for functional evaluation. Multiple organizations and private companies have acquired thousands of cells derived from control and diseased donors; many of these could be made available for TCs, either for research or testing purposes, through transfer agreements established through the repository. Further, contracts associated with cell repositories may require technical support and the incorporation of biomarkers and reporters into cells. Relatedly, it is important that the potential impact of reporters or biomarkers on cell function be evaluated and documented; again, input from regulatory agencies regarding approval of biomarkers in cell lines will be critical. If the tissue chip technology is to be developed for personalized medicine, standardized protocols for cell differentiation should be established, and procedures for distribution and technical support established. Contracts must be flexible and modifiable to allow inclusion of additional cell types as they are needed. Commercial repositories could be managed with ongoing input from academic scientists, the biotechnology sector, and the pharmaceutical industry to define current requirements.

With the establishment of resources such as cell repositories and databases for the TC Consortium, ethical and legal issues must be considered. As cell repositories are established, investigators and contractors must consider issues such as informed consent, donor permission, IRB approval, and permission/licensing issues related to use of de-identified cells. As databases are established, the need to facilitate information exchange must be balanced with the need for security (i.e., donor confidentiality, access privileges) and protection of intellectual property. For example, the presentation of information via website, even if access is limited, can adversely impact subsequent patent protection.

A strength of NCATS is the ability to mediate partnerships by facilitating introductions with the private sector and to foster such collaborations, which could benefit the TC Consortium. When appropriate, open sessions may be incorporated as a component of TC Investigator meetings; these sessions might be announced to attract partners, stakeholders and potential investors. Also, workshops focused on the status of MPS, regulatory agency insight and industry perspectives are encouraged. International interactions with NCATS related to tissue chip development may be particularly important given pressing European Union requirements related to animal testing and research efforts to develop alternative approaches [23]. The NIH might also organize symposia or other presentations at national scientific meetings on MPS that are relevant to different scientific disciplines. Such symposia are likely to attract the attention of researchers, clinicians and industry, and facilitate strategic collaborations.

## 4 Scenario for commercialization

In summary, responses solicited by the NCATS' *Request for Information* identify several approaches to TC public–private partnerships: 1) individual investigators partnering with biotechnology firms to develop an individual organ on a chip, 2) partnerships for the development of specific resources needed by the Tissue Chip Consortium (e.g., cell lines, microfluidic components, perfusion media), and 3) private sector maintenance of renewable resources such as cell repositories, data bases, or standardized protocols. In addition to coordinating the efforts of TC Consortium members, it is suggested that the NIH can facilitate public–private partnerships via subcontracts on individual grant awards or cooperative agreements. The NIH can also attract small business concerns through its Small Business Innovation Research (SBIR) and Small Business Technology Transfer (STTR) grants and contracts.

To attract private partners, an investigator will need a minimal, but viable technology for which there is some proof of concept, and a demonstrated commitment to development. A system or technology that is not yet validated or FDA-approved is not necessarily a roadblock to partnership; many smaller private sector entities are willing to contribute towards development to move the technology forward for SBIR/STTR funding, and eventual submission to the FDA. Initial funding of a partnership may be through government support (i.e., subcontract, SBIR/ STTR programs) or angel investors. At this stage it is important to identify a user market and to develop a marketing strategy; as development proceeds, the strategy may be modified in response to demands. With input from regulatory agencies, work should proceed towards product validation, adoption for use, FDA biomarker qualification or IND/NDA approval. This may be an iterative process involving further research and development, during which a publically funded investigator who is developing an organ system platform will benefit by the data generated and published, while a private sector partner will benefit by increased product visibility. Focus should then be on further refinement of the business plan, identifying and attracting investors appropriate for the potential revenue flow. If successful, the market share might be increased by modifications or further refinement of the platforms to expand their utility, diversification of the product line, or extending the network of contacts (e.g., international cooperation might be a consideration).

## 5 Partnerships associated with the Tissue Chip program

Clearly, both public and private partners can benefit from the more rapid development of organ system platforms and achieving their collaborative aims. Communication with regulatory agencies will provide additional benefit by helping to identify specific goals for further refinement and validation of tissue chip technology. Input from all stakeholders will be needed to move the TC Program beyond the development of individual and integrated MPS towards defined context of use, standardization and validation. Therefore future partnerships will require the inclusion of broad and complementary expertise from various scientific and professional disciplines to assure access to and easy adoptability of the tissue chips for translational scientific (e.g., disease modeling) and clinical use (e.g., personalized or precision medicine).

NCATS has begun to lay the groundwork for such partnerships to facilitate adoption of the organ system platforms as relevant models in biomedical research. Material Transfer Agreements (MTAs) with pharmaceutical companies are to provide TC Consortium Investigators with access to proprietary compounds, including those that failed in development due to toxicity in vivo. The next step will be to determine if the organ systems can replicate responses previously observed in vivo. Furthermore, Memorandums of Understanding (MOU) were executed, allowing pharmaceutical and small industry representatives to interact with Tissue Chip researchers regarding the use and marketing of current MPS technology. These interactions are crucial to facilitate feedback from potential stakeholders and to identify technical requirements early. Insights resulting from these interactions will help to redefine microphysiological systems as valid research tools and to make then widely available to the scientific community.
